# Influence of Klotho Protein Levels in Obesity and Sarcopenia: A Systematic Review

**DOI:** 10.3390/ijms26051915

**Published:** 2025-02-23

**Authors:** Diana G. Ariadel-Cobo, Brisamar Estébanez, Elena González-Arnáiz, María Pilar García-Pérez, Marta Rivera-Viloria, Begoña Pintor de la Maza, David Emilio Barajas-Galindo, Diana García-Sastre, María D. Ballesteros-Pomar, María J. Cuevas

**Affiliations:** 1 Institute of Biomedicine (IBIOMED), University of León, 24071 León, Spain; dgariadel@saludcastillayleon.es (D.G.A.-C.); b.estebanez@unileon.es (B.E.); egonzalezar@saludcastillayleon.es (E.G.-A.); mrivv@unileon.es (M.R.-V.); 2 Department of Endocrinology and Nutrition, Complejo Asistencial Universitario de León (CAULE), 24071 León, Spain; pgarciapere@saludcastillayleon.es (M.P.G.-P.); bpintor.asitec@saludcastillayleon.es (B.P.d.l.M.); dabarajas@saludcastillayleon.es (D.E.B.-G.); dgarciasastre@saludcastillayleon.es (D.G.-S.)

**Keywords:** adipose tissue, FGF, inflammaging, Klotho, sarcopenic obesity

## Abstract

The *Klotho* gene is recognized for its anti-aging properties. Its downregulation leads to aging-like phenotypes, whereas overexpression can extend lifespan. Klotho protein exists in three forms: α-klotho, β-klotho and γ-klotho. The α-klotho has two isoforms: a membrane-bound form, primarily in the kidney and brain, and a secreted klotho protein present in blood, urine, and cerebrospinal fluid. Klotho functions as a co-receptor for fibroblast growth factor-23 (FGF23), regulating phosphate metabolism. The membrane-bound form controls various ion channels and receptors, while the secreted form regulates endocrine FGFs, including FGF19 and FGF21. The interaction between β-klotho and FGF21 in muscle is critical in the development of sarcopenic obesity. This systematic review, registered in PROSPERO and conducted following PRISMA guidelines, evaluates klotho levels in individuals with obesity or sarcopenic obesity. The study includes overweight, obese, and sarcopenic obese adults compared to those with a normal body mass index. After reviewing 713 articles, 20 studies were selected, including observational, cross-sectional, cohort studies, and clinical trials. Significant associations between klotho levels and obesity, metabolic syndrome (MS), and cardiovascular risk were observed. Exercise and dietary interventions positively influenced klotho levels, which were linked to improved muscle strength and slower decline. Klotho is a potential biomarker for obesity, MS, and sarcopenic obesity. Further research is needed to explore its mechanisms and therapeutic potential.

## 1. Introduction

Klotho has been identified as an anti-aging gene as its downregulation leads to aging-like phenotypes [1] and extends lifespan when overexpressed [2]. After the discovery of α-klotho (*Klotho* gene (*KL* gene), chromosome 13q12.1) [2,3], two homologous proteins were identified and named β-klotho (*β-Klotho* gene, chromosome 4q26) [4,5] and γ-klotho (*KL* gene, chromosome 15q22.31) [6]. The α-klotho has two isoforms [7], as a membrane protein mainly expressed in distal convoluted tubules in the kidney and choroid plexus in the brain, but also in endocrine organs [8,9], and as a secreted klotho protein (s-klotho)—the extracellular domain—that locates in the blood, urine, and cerebrospinal fluid [10,11]. Research studies have acknowledged α-klotho as a co-receptor for fibroblast growth factor-23 (FGF23) since the phenotypes of mice lacking klotho and FGF23 were similar [12]. FGF23 and α-klotho might function in a common endocrine system that regulates phosphate metabolism [13]. Under physiological concentrations, FGF23 activation of the c-splice isoforms of FGFR1-3 and FGFR4 requires the formation of a binary complex with membrane-bound klotho, which enhances receptor affinity for FGF23 [5,14,15]. However, secreted klotho seems to be involved in the regulation of multiple ion channels, such as transient receptor potential cation channel, subfamily V, member 5 (TRPV5) [16], and growth factor receptors, such as insulin-like growth factor-1 (IGF-1) receptor [10], on the cell surface. In addition to klotho-interacting FGF23, there are two other endocrine FGFs that interact with β-klotho, which is mainly expressed in the liver and white adipose tissue: FGF19 and FGF21 [5,17]. Interestingly, while the intestine has been shown to secrete FGF19 to suppress liver bile acid synthesis in response to feeding, the liver has been found to secrete FGF21 to promote lipolysis and other metabolic responses in white adipose tissue as a consequence of fasting [18]. Therefore, klotho proteins could be principal actors in the regulation of endocrine FGFs [19]. FGF21 has also been found to be expressed in human muscle in response to hyperinsulinemia [20], so it has been proposed as a novel insulin-stimulated myokine [21]. In fact, endocrine-acting FGF21 released by muscle leads to a browning of white adipose tissue [22]. Thus, there is a constant intercommunication between white adipose and muscle tissues that can be altered with the age-dependent klotho and FGF21 decrease, which could be key in the development of sarcopenic obesity.

Overall, this work aims to systematically evaluate and synthesize the existing evidence on klotho levels in adults with obesity and sarcopenic obesity and to compare these levels with those of healthy adults. Additionally, it seeks to determine the existence of correlations between klotho levels and the presence of obesity or sarcopenic obesity.

## 2. Materials and Methods

**Figure 1:**
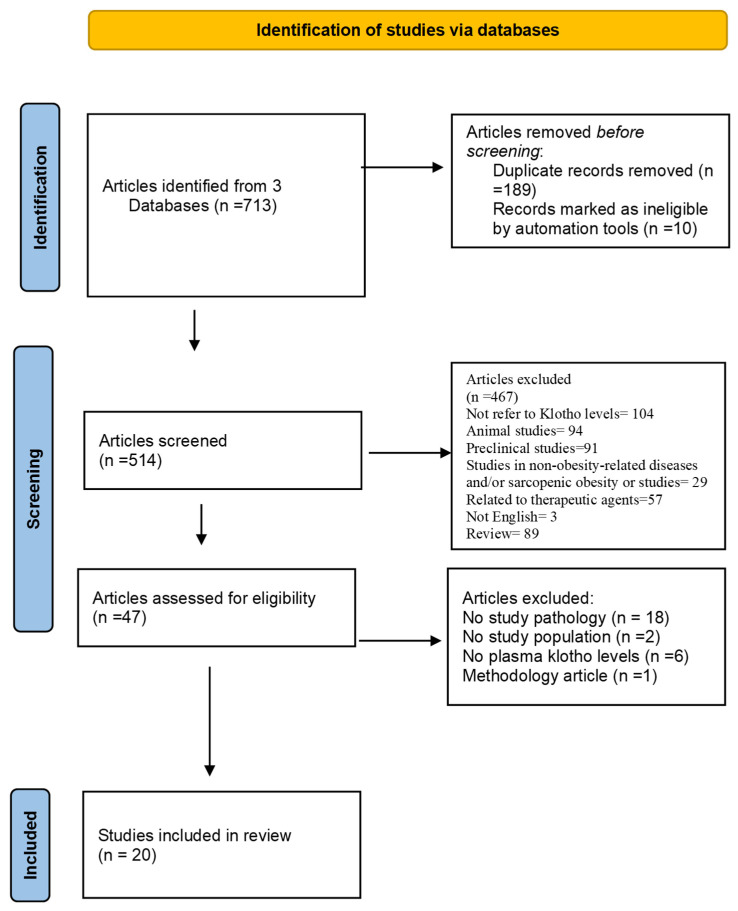
PRISMA flow diagram: study selection process for a systematic review on klotho protein levels in obesity and sarcopenic obesity [23].

This systematic review was registered at the PROSPERO international prospective register of systematic reviews (CRD42024569938). It was conducted following the Preferred Reporting Items for Systematic Reviews and Meta-Analyses (PRISMA) guidelines (Figure 1) [23]. It considers the findings of the clinical trials and below will clarify the systematic review question and PICOTS, study eligibility, search strategy, data collection and extraction, and validity assessment of risks of bias in included studies. No ethical approval was required for this study.

### 2.1. Systematic Review Question and PICOTS

This systematic review was conducted to investigate klotho levels in people with obesity or sarcopenic obesity. For the PICOTS of the review [population (P), intervention (I), comparison (C), outcome (O), time (T), and study design (S)], the criteria were defined prior to the literature search and are detailed in Table 1. Concisely, our study question was: Adults with overweight, body mass index (BMI) ≥ 25 Kg/m^2^ or obesity (BMI ≥ 30 Kg/m^2^) and adults with sarcopenic obesity (according to ESPEN EASO criteria) [24] (P); “intervention” is not directly applicable, as we were observing existing conditions, not interventions (I); compared with adults with normal BMI (C); may have an impact on klotho levels (O); at any time and for any duration (T); in clinical trials (CTs) (S).

### 2.2. Data Sources and Search Strategy

#### 2.2.1. Study Design, Literature Search, and Data Collection

Our objective was to compile the most important literature that provides evidence of klotho levels in the population with obesity and/or sarcopenic obesity and the difference between these levels and a healthy population. For a more comprehensive analysis, the authors included populations related to obesity, such as metabolic syndrome (MS). Additionally, the effect of physical exercise and diet interventions on klotho levels was examined.

No date restrictions were applied during the literature search. The bibliographic search was carried out mainly using the PubMed, Scopus, and Web of Science databases, as well as the reference lists of the selected studies, and included only manuscripts written in English. The titles and abstracts of all electronic articles were screened for eligibility.

Our search was conducted using the following MeSH/keywords: [“Klotho” AND (“sarcopenic obesity” OR “obesity” OR “sarcopenia”)]. Two authors (D.A. and E.G.) independently reviewed all articles for eligibility, while potential disagreements were resolved by consensus among all authors.

We searched for human studies, experimental studies, randomized controlled trials (RCTs), non-randomized studies of interventions, systematic reviews, and meta-analyses published in medical journals before 29 February 2024, while excluding case reports, editorials, conference abstracts, reviews, and posters.

#### 2.2.2. Data Extraction, Analysis, and Synthesis

All authors participated in the data analysis and each of them extracted data from each article in the preliminary stage. Two authors then reviewed all extracted data and added significant findings if any had been omitted. A wide variety of results were found. We focus on the following:

1. Klotho levels in a population with overweight, obesity, and/or sarcopenic obesity.
2. The differences in klotho levels compared with a healthy population.
3. The relationship between klotho levels and anthropometric parameters and between klotho levels and body composition.
4. The relationship between klotho levels and muscle strength parameters.
5. Populations related to obesity such as MS and polycystic ovary syndrome (PCOS) were included.

We noted results from studies that matched specific sections. Studies with similar results were tabulated together according to their sequence of description in the article.

Studies that did not refer to klotho levels, animal studies, preclinical studies, studies on non-obesity-related diseases and/or sarcopenic obesity, or studies related to therapeutic agents were excluded during the screening phase as being outside the scope of the review.

Among the 713 published articles considered, 189 were excluded after the removal of duplicates, and 477 were excluded during the selection phase based on the following exclusion criteria: failure to address the pathology under study, lack of relevance to the target population, absence of klotho level measurements, and limitations in the methodological approach.

All 47 full-text articles were assessed for eligibility, and 27 were excluded after the selection of abstracts or full text. Finally, 20 studies were selected. The flow chart of the selection process is presented in Figure 1.

## 3. Results

The analysis comprised twenty studies, which included one observational study, thirteen cross-sectional studies, two longitudinal cohort studies, and three clinical trials.

Among the six studies that established a relationship between klotho levels and obesity, three were related to MS, three were related to cardiovascular risk, four were related to physical exercise and diet, and four were related to older adult patients.

### 3.1. Studies Relating Klotho Levels with Obesity

Numerous studies have robustly linked klotho levels to obesity. Research by Amitani et al., Amaro-Gahete et al., Collin et al., Huang et al., Orces et al., and Bednarska et al. has all contributed to this understanding [25,26,27,28,29,30]. Table 1 presents the studies and results regarding klotho levels and their correlation with obesity, overweight, and/or sarcopenic obesity.

A comparative cross-sectional study of 34 adults (11 with obesity, 12 with restrictive anorexia nervosa [r-AN], and 11 controls) analyzed plasma α-klotho levels in different nutritional states. Amitani et al. (2013) [25] found lower α-klotho levels in obesity and r-AN, with a significant increase after BMI recovery in r-AN patients, suggesting klotho as a potential biomarker of nutritional status.

Amaro-Gahete et al. [26] conducted a cross-sectional study on 74 sedentary middle-aged adults (53.7 ± 5.1 years, 52.7% women) to examine the association between body composition and s-klotho plasma levels. The study identified significant positive correlations between BMI and s-klotho (β = 33.981, R^2^ = 0.125, *p* = 0.002) and between lean mass index (LMI) and s-klotho (β = 74.794, R^2^ = 0.346, *p* < 0.001). Both associations remained significant after adjusting for age, sex, and fat mass index, reinforcing the potential link between s-klotho and body composition parameters.

Bednarska et al. [27] analyzed serum β-klotho, FGF19, and FGF21 in 85 young, normal-weight women 67 with PCOS and 18 controls, finding significantly higher levels in PCOS patients. Strong correlations with PCOS diagnosis suggest these biomarkers may serve as potential predictors.

Huang et al. [28] conducted a cross-sectional study on 1950 adults (1119 men, 831 women) aged ≥ 40 years, using data from the National Health and Nutrition Examination Survey (NHANES) 2007–2016 to examine s-klotho concentrations, sagittal abdominal diameter (SAD), and metabolic parameters. The study identified a significant inverse association between SAD and s-klotho (β = −12.02), with a stronger negative correlation in individuals with BMI ≥ 30 Kg/m^2^ (β = −18.83, *p* = 0.001). Findings suggest a concentration-dependent relationship between SAD and s-klotho.

Orces (2022) [29] conducted a cross-sectional study on 4971 adults aged ≥ 40 years using NHANES 2007–2016 and confirmed lower s-klotho levels in obese individuals compared to those with normal weight, particularly in women. The study indicated an inverse association between general and abdominal obesity in women and s-klotho levels. Women who developed obesity earlier in life (765.0 pg/mL 25 years before, and 757.4 pg/mL 10 years before) had significantly lower mean s-klotho levels compared to those who were never obese (820.5 pg/mL). However, no significant differences in serum klotho levels were observed among men regardless of weight history.

Collins et al. [30] conducted an RCT involving 383 adults (BMI: 25–40 Kg/m^2^; age: 18–55 years) to evaluate changes in circulating α-klotho levels following weight loss interventions. Participants were randomly assigned to either a diet-only or a diet-plus-exercise program for 6 or 12 months and categorized based on their weight loss response: Responders (≥10% weight loss) and Non-responders (<5% weight loss) at both time points. Changes in circulating α-klotho levels were inversely correlated with reductions in weight (rs = −0.195), BMI (rs = −0.196), fat mass (FM) (rs = −0.184), and waist circumference (rs = −0.218), all of which were statistically significant (*p* < 0.05).

### 3.2. Studies Relating Klotho Levels with Metabolic Syndrome

Lower S-KL levels are linked to MS and its components [31,32], a phenomenon also observed in patients with human immunodeficiency virus (HIV) due to the inflammation and insulin resistance they exhibit [33]. Table 2 summarizes studies examining KL levels and their association with MS patients.

In research conducted by Cheng et al. [31], a study analyzed data from 9976 participants aged 18 and older (32.2% women), followed by Orces et al. [32], who studied 5069 participants (50% women, 57.4 ± 10.6 years). Both studies consistently demonstrated a negative relationship between the occurrence of MS and the concentrations of s-klotho. Even after accounting for various factors, the studies showed an inverse correlation between s-klotho levels and the number of MS components.

Detailed statistical analyses indicated that s-klotho levels were negatively associated with abdominal obesity and elevated triglycerides (TG) levels in both studies. Furthermore, a positive correlation was identified between s-klotho levels and high glucose concentrations in both investigations.

In a population of patients with HIV infection (n = 261), a study by Gutiérrez-Pérez et al. [33] found that the prevalence of MS was notably higher compared to a control group. Despite comparable weight and BMI between the HIV-infected and non-HIV groups, the HIV-infected population exhibited lower levels of β-klotho. This disparity implies that inflammation, heightened insulin resistance, and the presence of MS are contributing factors to the reduced β-klotho levels observed in HIV-infected individuals [33].

### 3.3. Studies Relating Klotho Levels with Cardiovascular Risk

Semba et al. [34], Amaro-Gahete et al. [35], and Lee et al. [36] demonstrated that higher s-klotho levels are associated with reduced cardiovascular and cardiometabolic risks, lower obesity rates, and improved lipid profiles. Table 2 provides an overview of studies investigating klotho levels and their association with cardiovascular risk.

In the study by Semba et al. [34] involving 1023 participants, 25.3% of whom had cardiovascular disease (55.1% women, aged 24–102 years), the median s-klotho concentration was 676 (530, 819) pg/mL. s-klotho correlated with age, high-density lipoprotein cholesterol (HDL-c), and C-reactive protein (CRP), but not with systolic blood pressure, fasting plasma glucose, or renal function. After adjusting for traditional cardiovascular risk factors, a significant association was found between log s-klotho and prevalent cardiovascular disease, with an odds ratio of 0.85 (95% confidence interval: 0.72 to 0.99) per one standard deviation increase.

Amaro-Gahete et al. [35] calculated a cardiometabolic risk score based on waist circumference, blood pressure, plasma glucose, HDL-c, and TG. This cross-sectional study included 214 healthy, sedentary adults, with approximately 64% women aged 18–25 years. A significant inverse relationship was found between s-klotho and the cardiometabolic risk score in middle-aged men and women (β = −0.658, R^2^ = 0.433, *p* < 0.001 and β = −0.442, R^2^ = 0.195, *p* = 0.007, respectively). However, no significant association was found between s-klotho and the cardiometabolic risk score in young, healthy adults (*p* > 0.5), nor for young, healthy men and women when analyzed separately (all *p* > 0.1).

In a study by Lee et al. [36] involving 13,154 participants, 75% being women aged 40–79 years, higher levels of circulating klotho were associated with lower rates of being overweight (β = −22.609, *p* = 0.0025) and obese (β = −23.716, *p* = 0.0011), as well as reduced rates of current smoking (β = −46.412, *p* < 0.0001) and alcohol consumption (β = −51.194, *p* < 0.0001). The study also found that s-klotho levels decreased with higher levels of TG (β = −0.117, *p* = 0.0006) and total cholesterol (β = −0.249, *p* = 0.0002).

### 3.4. Studies Relating Klotho Levels with Exercise and Diet

Fat oxidation and plasma s-klotho levels are positively correlated [37], while exercise [38] and dietary interventions [39] enhance s-klotho concentrations, improving metabolic and inflammatory profiles in sedentary and overweight individuals [40]. Table 3 presents a summary of studies exploring the relationship between klotho levels, diet, and exercise.

The study by Amaro-Gahete et al. [37] in 2019 aimed to explore the relationship between basal metabolic rate (BMR), fuel oxidation, and plasma s-klotho in 74 middle-aged sedentary adults (53% women, 53.7 ± 5.1 years). The results revealed no significant correlation between BMR and plasma s-klotho (*p* > 0.1). However, both basal fat oxidation and maximal fat oxidation (MFO) during exercise exhibited positive associations with s-klotho (both *p* < 0.001), which remained significant even after adjusting for age, sex, and FM. Interestingly, there were no significant associations found between BMR, basal fat oxidation, or MFO and chronological age (all *p* > 0.1). These findings suggest a strong link between basal fat oxidation, MFO, and plasma s-klotho in middle-aged sedentary adults.

Amaro-Gahete et al. [38] investigated the impact of different training modalities on s-klotho plasma levels in 68 sedentary, middle-aged adults (52.7% women, 53.4 ± 5.0 years). The study revealed that s-klotho levels increased in response to physical activity interventions like physical activity recommendations, high-intensity interval training (HIIT), and HIIT combined with whole-body electromyostimulation (HIIT-EMS) compared to the control group (*p* = 0.003, *p* = 0.019, *p* < 0.001, respectively), with no significant differences between the intervention groups. Positive associations were found between changes in LMI and s-klotho levels, while negative associations were observed between changes in FM outcomes and s-klotho levels, persisting even after adjusting for sex and age.

In the cross-sectional study conducted by Ma et al. [39], which used data from the NHANES from 2007 to 2016, including 8456 participants aged 40–79 years, data from 24-h dietary recalls were used to calculate the Healthy Eating Index 2015 (HEI-2015) for each participant. A positive correlation was observed between HEI-2015 and s-klotho plasma levels (β = 0.74, 95% CI: 0.21, 1.27, *p* = 0.0067). The analysis indicated a turning point of HEI-2015 at 45.15, where a significant dose-response relationship was observed between HEI-2015 and s-klotho levels. Furthermore, individuals with a normal BMI showed a more pronounced association between HEI-2015 and s-klotho concentrations.

Silva-Reis et al. [40] explored the effects of 12 weeks of combined physical exercise on lung function and mechanics in non-obese (n = 12), overweight (n = 17), and obese grade I (n = 11) women. The study showed that the exercise regimen reduced pro-fibrotic IGF-1 levels in overweight and obese groups, increased klotho levels in obese individuals, and decreased exhaled nitric oxide levels in both overweight and obese groups.

### 3.5. Studies Relating to Klotho Levels in Older Adults

Lower s-klotho levels are associated with reduced grip and knee strength, highlighting its role in muscle function [41,42]. However, studies report no significant links between s-klotho and frailty or fractures, warranting further investigation [43,44]. Table 4 presents a summary of studies examining klotho levels and their associations in older adults.

In the Aging in the Chianti Area (InCHIANTI) study [41], involving 804 older adults aged 65 and above (55.8% women), grip strength showed a positive correlation with s-klotho at a threshold of less than 681 pg/mL. After adjusting for several factors, s-klotho (per 1 standard deviation increase) was associated with grip strength (β = 1.20, SE = 0.35, *p* = 0.0009) in adults with s-klotho levels below 681 pg/mL, indicating that older adults with poorer skeletal muscle strength have lower s-klotho levels.

Semba et al. [42] analyzed the relationship between s-klotho levels and knee strength in older adults (aged 71–79 years) based on data from 1983 participants. Individuals in the highest tercile of s-klotho exhibited significantly greater knee extension strength (β = 0.72, SE = 0.018, *p* < 0.0001) than those in the lowest tercile, after adjusting for various factors in a multivariable linear regression model. Additionally, participants in the highest tercile of s-klotho at baseline experienced less decline in knee strength over 4 years of follow-up (β = −0.025, SE = 0.011, *p* = 0.02) compared to those in the lowest tercile.

The study by Chalhoub et al. [43] found no significant association between the lowest quartile of s-klotho levels and non-spine, hip, or vertebral fractures. This analysis included 2776 participants aged 70–79 from the Health ABC cohort, of whom 52% were women.

Polat et al. [44] conducted a cross-sectional study to examine the relationship between frailty and s-klotho levels in geriatric patients. The study included 89 individuals aged 65 and older, divided into two groups: 45 frail patients and 44 non-frail controls. The mean s-klotho levels of the control and frail groups were 0.76 ± 1.01 ng/mL and 0.54 ± 0.61 ng/mL, respectively. However, there was no statistically significant difference between the two groups (*p* = 0.286).

## 4. Discussion

Obesity is a major public health challenge, with a rising prevalence affecting one in eight individuals worldwide [45]. Beyond classical metabolic complications, obesity frequently coexists with sarcopenia, a condition characterized by the loss of muscle mass and functionality, leading to sarcopenic obesity. This phenotype exacerbates metabolic dysfunction, increases the risk of disability, and is associated with higher mortality rates [46].

Epidemiological studies indicate that sarcopenia affects approximately 10–16% of older adults globally [47]. In Spain, no nationwide epidemiological studies have been conducted, and prevalence varies by setting, affecting between 15% and 50% [48,49,50,51,52]. Additionally, the EXERNET multicenter study underscores the relevance of body composition assessment in aging populations [53]. Sarcopenic obesity arises from a multifactorial interaction between adipose tissue dysfunction, systemic inflammation, and muscle degradation, further intensifying insulin resistance and mitochondrial dysfunction [54].

Several biomarkers have been identified for sarcopenia, including the creatinine/cystatin C ratio, C-terminal agrin fragment, and multiple microRNAs (miR-7a-1-3p, miR-135a-5p, miR-151-5p, miR-196b-5p) [55,56,57]. Additionally, inflammatory and metabolic markers such as CRP, interleukin-6 (IL-6), tumor necrosis factor-alpha, IGF-1, myostatin, follistatin, and growth differentiation factor 15 have been highlighted [58,59,60]. While these biomarkers exhibit potential for sarcopenia diagnosis, their diagnostic accuracy remains limited, necessitating further studies to validate their clinical applicability. Despite its clinical significance, no pharmacological treatments for sarcopenic have been approved [61]. Investigational strategies include myostatin inhibitors, follistatin, IGF-1, and inflammatory modulators such as CRP and IL-6 [62].

Klotho, a pleiotropic protein associated with longevity, has emerged as a critical factor in aging research [63]. Its deficiency has been linked to various age-related diseases, including cancer, chronic kidney disease, ataxia, diabetes, and skin atrophy [64,65]. However, the relationship between klotho and conditions such as obesity and MS remains incompletely understood, providing the basis for this systematic review.

This analysis, encompassing 20 studies, consistently revealed a negative association between s-klotho levels and obesity, particularly in women. Notably, women who developed obesity earlier in life exhibited significantly lower s-klotho levels compared to those with normal weight [25,26,27,28,29,30]. This observation underscores the possibility that early-life obesity may lead to long-lasting biological changes impacting klotho expression, with potential sex-specific implications. These findings highlight the need to further explore the mechanistic links between obesity and longevity-related pathways mediated by klotho.

Similarly, research on school-age children revealed that serum α-klotho concentrations were negatively associated with obesity-related parameters, particularly in girls, indicating that early-life obesity might have a more pronounced effect on klotho levels in females [66]. Moreover, in patients with type 2 diabetes mellitus, a condition often associated with obesity, lower serum klotho levels were observed in those with moderate cognitive impairment, further linking metabolic health and klotho expression [67].

The review also demonstrated that s-klotho levels are inversely associated with specific components of MS, such as abdominal obesity and elevated TG levels, while a positive association was observed with elevated glucose levels [31,32]. This suggests that s-klotho might serve not only as an early biomarker for metabolic risk but also as a mediator in the development and progression of metabolic disorders. Future research should prioritize elucidating the underlying mechanisms to determine whether klotho could be leveraged as a therapeutic target.

The evidence indicates that the concentration of the klotho protein might influence the onset and advancement of MS. This underscores the significant role that klotho could have in maintaining metabolic health, particularly regarding its relationship with conditions such as obesity, lipid imbalances, and elevated blood sugar levels [67]. This is consistent with the findings presented in the research by de Luca Corrêa et al. [68].

Regarding cardiovascular risk, higher circulating klotho levels have been associated with lower rates of overweight and obesity, as well as reduced prevalence of smoking and excessive alcohol consumption [34,35,36]. Moreover, in individuals with carotid atherosclerosis, lower klotho protein levels in the blood and reduced *KL* gene expression in vascular tissues were observed alongside higher carotid-intima media thickness values [69]. These findings suggest that klotho may play a role in mitigating cardiovascular risk through its anti-inflammatory and vascular-protective properties [70].

Lifestyle interventions such as regular aerobic exercise [37,38,39,40] and healthy dietary patterns [39] appear to support the maintenance or increase of s-klotho levels. These interventions may mitigate age-related metabolic and inflammatory changes, thereby preserving functional health. Additionally, elevated s-klotho levels have been positively correlated with muscle strength and physical performance in older adults, such as increased knee extension strength and reduced strength decline over time [41,42,43,44]. These findings underscore the potential of klotho in preventing functional deterioration and promoting healthy aging.

Despite these promising insights, several limitations must be acknowledged. The variability in study designs, including observational, cross-sectional, and cohort studies, complicates direct comparisons and reduces the generalizability of findings. Gender-related discrepancies, as reported in Orces’ study [29], further highlight the need for more targeted research to understand sex-specific variations. Additionally, the heterogeneity of study populations, including those with conditions like PCOS, r-AN, and older adults, warrants cautious interpretation of the results.

The primary limitation of the review lies in the establishment of associations rather than causative relationships between klotho levels and obesity-related parameters. Cross-sectional studies, which provide only a snapshot in time, are insufficient to infer causality or directionality in these associations. Furthermore, variations in adjustments for confounding factors across studies and potential sample size limitations could introduce bias and affect the robustness of conclusions.

## 5. Conclusions

The review uncovered a complex relationship between klotho protein levels and various aspects of obesity and related health issues. Higher levels of s-klotho have been positively linked with BMI and LMI, indicating a possible association between klotho levels and body composition. Lower concentrations of s-klotho have been found in obese women compared to those with normal weight, suggesting potential gender disparities in klotho expression related to obesity. Inverse associations have been observed between s-klotho concentrations and the prevalence of MS and cardiovascular risk, positioning s-klotho as a potential predictive biomarker for these conditions. Exercise and diet have been shown to impact klotho levels, with exercise interventions leading to increased s-klotho levels, emphasizing the significance of lifestyle in obesity management. Higher klotho levels have been associated with greater muscle strength in older adults, hinting at a protective role against age-related declines in muscle function. Implementing klotho level testing in clinical settings could aid in assessing obesity risks and monitoring the effectiveness of intervention strategies. Further research is required to clarify the mechanisms through which klotho influences metabolic processes and its potential therapeutic applications.

## Figures and Tables

**Table 1:** Study characteristics and patient demographics in obesity and sarcopenic obesity research.

Study	Patient Type *n*	Groups (n)	Age (years)	Sex	BMI (Kg/m^2^)	Body Composition	Strength Parameter	Parameter and Measuring Method, (Unit.). Level
Amitani et al., 2013 [25] Cross-sectional study	r-AN—Obesity 2013 Women *n* = 34	r-AN 12 O 11 CG 11	r-AN 21.0 ± 1.65 O 21.3 ± 0.95 CG 21.0 ± 1.29	N/A	r-AN 13.12 ± 0.26 O 35.72 ± 3.17 CG 21.84 ± 0.36 *p* = <0.001	N/A	N/A	S-Klotho, ELISA, (pg/mL) r-AN 764.64 ± 65.43 O 847.09 ± 111.31 CG 1391.62 ± 144.96 *p* = <0.01
Amaro-Gahete et al., 2018 [26] Cross-sectional study	FIT-AGING study. 40–65 years *n* = 74	BMI (Kg/m^2^) NW ≥ 18.5–<25 OW ≥ 25–<30 O ≥ 30	53.7 ± 5.1	M (n) 35	27.7	DEXA LM (Kg) 43.5 ± 11.7 LMI (LM/m^2^) 43.5 ± 11.7 FMI (FM/m^2^) 10.7 ± 3.1 FM (Kg.%) 30 ± 8.4, 3.9 ± 9.1	N/A	S-Klotho, ELISA, (pg/mL). NW = 988.08 O = 668.43 *p* = 0.033
Bednarska et al., 2020 [27] Cross-sectional study	PCOS 2020 18–40 years *n* = 67	PCOS 49 CG 18	PCOS 25.85 ± 5.22 CG 27.78 ± 5.6	N/A	PCOS 26.55 ± 6.92 CG 21.35 ± 2.08	N/A	N/A	β-Klotho, ELISA, (pg/mL) NW 8695.1 ± 3484.5 OW 9287.5 ± 3337 O 7734.7 ± 4183.5 *p* = 0.427
Huang et al., 2022 [28] Cross-sectional study	NHANES 2011–2012 40–79 years *n* = 9756	QSAD (cm) Q1 < 20.4 Q2 20.4 to <23.2 Q3 23.2 to <26.1 Q4 > 26.1	Q1 52.66 ± 9.54 Q2 56.36 ± 10.31 Q3 57.34 ± 10.65 Q4 58.20 ± 10.49 *p* < 0.001	M (%, n) Q1 27.3, 35 Q2 39.2, 187 Q3 54.0, 415 Q4 53.3, 494	Q1 21.15 ± 2.03 Q2 24.15 ± 2.39 Q3 27.52 ± 2.87 Q4 34.78 ± 6.05 *p* < 0.001	N/A	N/A	S-Klotho, ELISA, (pg/mL) Q1 956.38 ± 303.37 Q2 911.56 ± 316.43 Q3 888.53 ± 324.32 Q4 877.95 ± 310.98 *p* = 0.029
Orces et al., 2022 [29] Cross-sectional study	NHANES 2013–2014, 2015–2016 40–79 years *n* = 4971	10 years and 25 years to baseline NO, ONO, NOO, AO	57.4 ± 10.6	(%, n) M 47.9, 2380	NW 23.8 OW 34.4 O 41.8	N/A	N/A	S-Klotho, ELISA, (pg/mL) NW 820.6, OW 765.1, O 772.8 *p* = <0.001
Collins et al., 2023 [30] Clinical trial	Heart Health Study 2011–2015 *n* = 152	DIET DIET + MVIPA150 DIET + MVIPA250	45.4 ± 8.0	N/A	32.1 ± 3.7	LM (Kg): R. 48.4, NoR. 47.2 FM (Kg): R. 39.3, NoR. 38.4 PBF (%): R. 44.8, NoR. 45 WC (cm): R. 107.3, NoR. 104.5	N/A	S-Klotho, ELISA, (pg/mL) Before the intervention R = 936.2 (870.6–1006.5) NoR = 926.1 (800.4–1077)

Abbreviations: AO, always obese; BMI, body mass index; CG, control group; DEXA, dual-energy X-ray absorptiometry; FMI, fat mass index; FM, fat mass; LM, lean mass; LMI, lean mass index; M, male; MVIPA150, moderate to vigorous intensity physical activity 150 min; MVIPA250, moderate to vigorous intensity physical activity 250 min; N/A, not applicable; NHANES, National Health and Nutrition Examination Survey; NO, never obese; NOO, non-obese to obese; No, non-responder; NW, normal-weight; O, obese; ONO, obese to non-obese; OW, overweight; PBF, percent body fat; PCO, polycystic ovary syndrome; Q, quartiles; QSAD, quartiles of sagittal abdominal diameter; R, responder; r-A, restricting-type anorexia nervosa; WC, waist circumference.

**Table 2:** Study characteristics and patient demographics in metabolic syndrome and cardiovascular risk research.

Study	Patient Type *n*	Groups (n)	Age (years)	Sex	BMI (Kg/m^2^)	Body Composition	Strength Parameter	Parameter and Measuring Method, (Unit.). Level
Semba et al., 2011 [34] Cross-sectional study	InCHIANTI study 2001–2003 24–102 years *n* = 1023	TK (pg/mL) T1 < 586 T2 587–769 T3 > 770	T1 75 (69–80) T2 72 (66–78) T3 72 (64–77)	M (%) Q1 47.9 Q2 46.5 Q3 40.2	Q1 26.3 (23.6–28.8) Q2 26 (23–28.6) Q3 26 (23.1–28.4)	N/A	LG (Kg): Q1 90.1, Q2 92.0, Q3 93.8 LG N-m/Kg: Q1 1.19, Q2 1.23, Q3 1.26 DN (Kg): Q1 0.9, Q2 0.40, Q3 0.41	S-Klotho, ELISA, (pg/mL) N/A
Amaro-Gahete et al., 2020 [35] Cross-sectional study	FIT-AGING and ACTIBATE study 40–65 and 18–25 years. *n* = 214	YA 145 OA 74	N/A	M (n) YA 42 OA 35	YA 25 OA 26.7	N/A	N/A	S-Klotho, ELISA, (pg/mL) YA 823.1 OA 775.3
Lee et al., 2022 [36] Observational study	NHANES 2007–2016 40–79 years *n* = 13,154	QK (pg/mL) Q1 < 654.6 Q2 654.6 to 802.4 Q3 802.4 to 993.3 Q4 ≥ 992.4	Q1 59.10 ± 11.11 Q2 57.94 ± 10.84 Q3 57.35 ± 10.73 Q4 56.35 ± 10.62 *p* = < 0.0001	M (%, n) Q1 26.32, 1669 Q2 26.87, 1704 Q3 24.50, 1554 Q4 22.29, 1414	N/A	N/A	N/A	S-Klotho, ELISA, (pg/mL) N/A
Cheng et al., 2022 [31] Cross-sectional study	NHANES 2007–2012 ≥18 years. *n* = 9976	MS 3906 CG 6070	MS 58.93 ± 10.4 CG 56.4 ± 10.83	M (%, n) MS 48.1. 1880 CG 50.3, 3053	MS 32.40 ± 6.29 CG 27.65 ± 5.85	N/A	N/A	S-Klotho, ELISA, (pg/mL) MS 848.35 ± 292.92 CG 871.54 ± 311.68 *p* = <0.001
Orces et al., 2022 [32] Cross-sectional study	NHANES: 2013–2014, 2015–2016 40–79 years *n* = 5069	MS 2279 CG 2290	57.4 ± 10.6	M (%, n) MS 44.8. 1080 CG 55.2, 1330	NW 23.8 O 34.4 MO 41.8	N/A	NA	S-Klotho, ELISA, (pg/mL) N/A
Gutiérrez-Pérez et al. [33] Observational study	CAPASITS SAIH Mexico HIV infection 18–50 years *n* = 261	WHIV: 179 NHIV: 82	WHIV: 39.21 ± 10.66 NHIV: 32.39 ± 10.56	M (%, n) WHIV 75.4, 135 NHIV 69.5, 57	WHIV: NW 21.9, O 29.7 NHIV: NW 22.3, O 30.8	N/A	N/A	β-Klotho, ELISA, (pg/mL) WHIV 4.05 ± 0.04 NHIV 4.19 ± 0.03 *p* = 0.011

Abbreviations: BMI, body mass index; CAPASITS, Center for Prevention and Ambulatory Care of AIDS and Sexually Transmitted Infections; CG, control group; DN, dynamometry; InCHIANTI study, Aging in the Chianti Area; LG, leg strength; M, male; MO, morbid obesity; MS, metabolic syndrome; NHANES, National Health and Nutrition Examination Survey; NHIV, non-HIV; N/A, not applicable; OA, older adult; O, obese; Q, quartiles; QK, quartiles klotho; T, tercile; T1, lowest tercile; T2, middle tercile; T3, highest tercile; WHIV, with HIV; YA, young adults.

**Table 3:** Study characteristics and patient demographics in exercise and diet research.

Study	Patient Type *n*	Groups (n)	Age (years)	Sex	BMI (Kg/m^2^)	Body Composition	Strength Parameter	Parameter and Measuring Method, (Unit.). Level
Amaro-Gahete et al., 2019 [37] Cross-sectional study	FIT-AGING study 40–65 years *n* = 74	N/A	53.7 ± 5.1	M (%, n) 47.35	26.7 ± 3.8	DEXA LM (Kg) 43.5 ± 11.7 LMI (LM/m^2^) 43.5 ± 11.7 FMI (FM/m^2^) 10.7 ± 3.1 FM (Kg, %) 30 ± 8.4, 39.9 ± 9.1	VO2max (mL/Kg/min) 30.5 ± 5.6	S-Klotho, ELISA, (pg/mL) 775.3 ± 363.7
Amaro-Gahete et al., 2019 [38] Clinical trial	FIT-AGING study 40–65 years *n* = 68	CG 15 PAR 17 HIIT 17 HIIT-EMS 19	CG 51.7 ± 4.1 PAR 54.9 ± 4.5 HIIT 53.5 ± 5.6 HIIT-EMS 53.5 ± 5.2	M (%, n) CG 40.6 PAR 47.1, 8 HIIT 47.1, 8 HIIT-EMS 52.6, 10.	CG 26.7 ± 3.9 PAR 25.4 ± 2.9 HIIT 26.4 ± 3.2 HIIT-EMS 28.6 ± 4.6	All LMI (LM/m^2^) 15.4 ± 2.8 FMI (FM/m^2^) 10.7 ± 3.1 FM (%) 39.6 ± 8.5	N/A	S-Klotho, ELISA, (pg/mL) B/PIT CG 922.5 ± 290.3/862.9 ± 364.4 PAR 714.3 ± 294.5/1055.4 ± 435.9 HIIT 788.5 ± 276.8/1057.1 ± 273.3 HIIT-EMS 808.5 ± 499.0/1259.7 ± 613.1 *p* = <0.001
Ma et al., 2022 [39] Cross-sectional study	NHANES 2007–2016 40–79 years *n* = 8456	QHEI-2015 Q1 ≤ 60 Q2 > 60, ≤70 Q3 > 70, ≤80 Q4 > 80	Q1 56.76 ± 10.98 Q2 58.49 ± 10.77 Q3 59.19 ± 10.93 Q3 59.98 ± 10.32	M (%, n) Q1 52.94, 3102 Q2 52.94, 3102 Q3 44.18, 338 Q4 44.62, 112	Q1 28.33 ± 6.31 Q2 27.80 ± 5.84 Q3 26.91 ± 5.19 Q4 26.06 ± 5.02	N/A	N/A	S-Klotho, ELISA, (pg/mL) Q1 851.64 ± 312.30 Q2 878.35 ± 329.71 Q3 879.80 ± 312.65 Q4 903.58 ± 332.19 *p* = <0.001
Silva-Reis et al., 2022 [34,40] Clinical trial	Combined physical exercise. Women 30–59 years *n* = 41	NW 12 OW 17 O 11	NW 43.5 ± 11.3 OW 47.35 ± 11.75 O 47.36 ± 10.64	N/A	NW 22 ± 1.9 OW 27.93 ± 1.67 O 31.98 ± 1.45	N/A	N/A	S-Klotho. ELISA, (pg/mL) Does not add value PRH vs. PoRH: NW < 0.0489, OW < 0.0333, O > 0.05

Abbreviations: B, baseline; BMI, body mass index; CG, control group; DEXA, dual-energy X-ray absorptiometry; FMI, fat mass index; FM, fat mass; HIIT, high-intensity interval training group; HIIT-EMS, high-intensity interval training adding whole-body electromyostimulation group; LM, lean mass; LMI, lean mass index; M, male; NW, normal-weight; N/A, not applicable; O, obese; OW, overweight; PAR, physical activity recommendations for adults proposed by the World Health Organization group; P, post-intervention; PoRH, post-rehabilitation; PRH, pre-rehabilitation; Q, quartiles; QHEI-2015, quartiles The Healthy Eating Index 2015; VO2max, maximum oxygen uptake.

**Table 4:** Study characteristics and patient demographics in older adults.

Study	Patient Type *n*	Groups (n)	Age (years)	Sex	BMI (Kg/m^2^)	Body Composition	Strength Parameter	Parameter and Measuring Method, (Unit.). Level
Semba et al., 2012 [41] Longitudinal cohort study	InCHIANTI study 2001–2009 >65 years *n* = 804	K (pg/mL), % >681, 46.8% <681, 53.2%	75 (71–80)	M (%, n) 44.2, 355	26.4 (23.7–28.7)	N/A	Grip strength (Kg) 26.5 (20.3–36.5)	S-Klotho, ELISA, (pg/mL) 664 (521–811)
Semba et al., 2016 [42] Cross-sectional study	Health ABC Study 70–79 years *n* = 1983	TK (pg/mL) T1 < 536 T2 536–747 T3 > 747	T1 74.5 T2 74.5 T3 74.5	M (%, n) T1 M 51.0 T2 M 52.0 T3 M 48.1	T1 27.4 T2 27.1 T3 27.0	N/A	Knee extensor strength (Kg): T1 90.1, T2 92.0, T3 93.8 Grip strength (Kg): T1 39, T2 40, T3 41 Knee extensor strength, mean/weight (N-m/Kg): T1 1.19, T2 1.23, T3 1.26 *p* = 0.002	S-Klotho, ELISA, (pg/mL) N/A
Chalhoub D et al., 2016 [43] Longitudinal cohort study	Health ABC Study 70–79 years *n* = 2776	QK (pg/mL) Every group, n = 694 Q1 320.6 to 437.3 Q2 521.6 to 592.1 Q3 670.1 to 756.2 Q4 887.7 to 1186.4	Q1 74.7 ± 2.9 Q2 74.8 ± 2.9 Q3 74.5 ± 2.8 Q4 74.6 ± 2.9	M (%, n) Q1 48.1, 334 Q2 52.9, 367 Q3 50.0, 347 Q4 44.5, 309	Q1 27.3 ± 4.8 Q2 27.3 ± 4.4 Q3 27.1 ± 1 Q4 27.1 ± 5.1	ALM (Kg) Q1 20.0 ± 5.0 Q2 20.0 ± 4.9 Q3 20.2 ± 4.9 Q4 20.2 ± 4.8	Grip strength (Kg): Q1 31.4 ± 10.5, Q2 31.7 ± 10.5, Q3 32.1 ± 10.6, Q4 32.2 ± 10.9 Gait Speed (m/seg): Q1 0.93 ± 0.47, Q2 0.96 ± 0.46, Q3 0.97 ± 0.46, Q4 0.95 ± 0.44	S-Klotho, ELISA, (pg/mL) N/A
Polat et al., 2020 [44] Cross-sectional study	Ankara University Faculty 2018 >65 years *n* = 89	CG 44 FG 45	FG 72.7 ± 4.45 CG 79.36 ± 6.91 *p* = <0.001	M (n) FG 14 CG 17	CG 30.53 ± 4.24 FG 27.76 ± 7.89 *p* = 0.003	N/A	Grip strength (Kg): CG 25.6 ± 6.71, FG 16.65 ± 6.83 *p* = < 0.001 Walking speed, m/s: CG 7.19 ± 0.98, FG 9.51 ± 2.95 *p* = < 0.001	S-Klotho, ELISA, (pg/mL) CG 0.76 ± 1.01 FG 0.54 ± 0.61 *p* = 0.286

Abbreviations: ALM, appendicular lean mass; BMI, body mass index; CG, control group; FG, frailty group; InCHIANTI study, Aging in the Chianti Area; K, klotho; M, male; Q, quartiles; QK, quartiles klotho; T, tercile; TK, tercile klotho; T1, lowest tercile; T2, middle tercile; T3, highest tercile.
